# Advantages of Sheep Infrapatellar Fat Pad *Adipose Tissue Derived Stem Cells* in Tissue Engineering

**DOI:** 10.15171/apb.2016.016

**Published:** 2016-03-17

**Authors:** Parviz Vahedi, Jafar Soleimanirad, Leila Roshangar, Hajar Shafaei, Seyedhosein Jarolmasjed, Hojjatollah Nozad Charoudeh

**Affiliations:** ^1^ Department of Anatomical Sciences, Faculty of Medicine, Tabriz University of Medical Sciences, Tabriz, Iran.; ^2^ Department of Anatomical Sciences, Maragheh Faculty of Medicine, Maragheh, Iran.; ^3^ Stem Cell Research Center, Faculty of Medicine, Tabriz University of Medical Sciences, Tabriz, Iran.; ^4^ Department of Clinical Sciences, Faculty of Veterinary Medicine, University of Tabriz, Tabriz, Iran.

**Keywords:** Anti-Human Antibodies, Infrapatellar fat pad adipose tissue, CD markers, ASCs

## Abstract

***Purpose:*** The goal of this study has been to evaluate adipose tissue derived stem cells (ADSC_s_) from infrapatellar fat pad and characterize their cell surface markers using anti-human antibodies, as adipose tissue derived stem cells (ADSC_s_) have great potential for cellular therapies to restore injured tissues.

***Methods:*** Adipose tissue was obtained from infrapatellar fat pad of sheep. Surface markers evaluated by flow cytometry. In order to evaluate cell adhesion, the Polycaprolactone (PCL) was sterilized under Ultraviolet (UV) light and about 1×10^5^ cells were seeded on PCL. Then, ASCs- PCL construct were evaluated by Scanning Electron Microscopy (Mira3 Te Scan, Czech Republic).

***Results:*** We showed that adipose tissue derived stem cells (ADSC_s_) maintain their fibroblastic-like morphology during different subcultures and cell adhesion. They were positive for CD44 and CD90 markers and negative for CD31 and Cd45 markers by human antibodies.

***Conclusion:*** Our results suggest that ASCs surface markers can be characterized by anti-human antibodies in sheep. As stem cells, they can be used in tissue engineering.

## Introduction

Tissue engineering, as a new approach to reconstruction and regeneration of damaged tissues, is used to repair the injured tissues.^1^ The adipose tissue can be considered as an attractive alternative source.^2^ It can be collected in large quantities from adipose tissue fragments. Adipose tissue derived stem cells (ADSCs) are an abundant cell source, applied in pre-clinical studies, and are well known owing to their capacity to undergo osteogenic, chondrogenic, adipogenic, neurogenic and myogenic differentiation in vitro.^3^ Furthermore, ASCs have been shown to be immune privileged, and more genetically stable in long-term culture, compared to BMSCs.^4^ The efficacy of *ASCs* for tissue regeneration is currently under assessment in clinical trials.^5^

Adipose tissue is well established as an easily accessible source of adult mesenchymal stem cells with properties suitable for tissue engineering and cell therapy. *ASCs* can differentiates into variety of cell types^6^ and, therefore, possess great potential favorable to cellular therapies to restore injured tissues. The presence of MSCs in the infrapatellar fat pad (IFP-MSCs) of the knee has been recently demonstrated. These cells can differentiate towards different mesodermal lineages and were used to treat the osteoarthritis (OA) in a rabbit model.^7^ It has been shown that MSCs derived from infrapatellar fat to possess significant chondrogenic potential.

The initial methods to isolate cells from adipose tissue were pioneered by Rodbell and colleagues in 1960_s_ using rat fat tissue. Onwards, the methods have been adapted by several other groups to encompass human tissues.^8^ The current methods of isolating ASCs consists in collagenase digestion followed by isolating stromal/vascular cells from primary adipocytes by centrifugal separation.^9^ Adipose tissue, as a source of stem cells, can be easily harvested in comparison to bone marrow. ASCs have potential benefits for tissue engineering applications, being simply isolated without painful procedures or site injury.^10^ In this study adipose tissue derived stem cells in sheep inferapatellar fat pad were characterized by anti-human antibodies; their cell adhesion and fibroblast–like morphology was evaluated for use in tissue engineering.

## Materials and Methods

### Isolation of adipose tissue stem cells 

Adipose tissue was obtained from Infrapatellar fat pad of 5 male sheep (Bergamasca–Massese) weighing 20-25 kg, aged 12 months in slaughterhouse under sterile condition. It was washed several times with sterile phosphate-buffered saline (PBS; Sigma, Germany) containing 1% penicillin/streptomycin to remove contaminating debris and red blood cells. It was minced finely using surgical scissor to many small pieces and treated with 500µl type1 collagenase (Gibco, Japan) for 45 minute. After digestion, it was centrifuged at 1,600 rpm for 10 min. The supernatant was discarded and cell pellets were suspended in 100µl medium (DMEM; sigma, Germany) containing 1% penicillin/streptomycin, 37 mg bicarbonate and 10% FBS.

### Culture of adipose tissue stem cells

Adipose tissue derived stem cells (ADSCs), about 1×10^5^cells were seeded in a T- 25 culture flask and incubated at 37°C and 5.0% CO_2_ in humidified incubator. The medium was replaced every 2 days. After reaching 80-90% confluency, the cells were detached with trypsin (sigma), and counted in a neubauer hemocytometer by the invert microscope. The culture of cells was expanded to passage six, and analyzed by flow cytometry. Cells’ appearance and morphometrical analysis were done by H&E staining and invert microscopy.

### Population doubling time (PDT)

Optimal density of ADSCs about 1×10^3^ cells/cm^2^ in a DMEM supplemented with 15% FBS were seeded into T25 culture flasks. When the ASCs reached 80-90% confluency, the cells were lifted and counted in a neubauer hemocytometer by an invert microscope. Using the following equation, cell’s PDT was calculated at passages through 1 to 6. Where, N_0_ is the initiating cell number, N is the final cell number, and C.T is the culture time.

$$\left(PDT\right)=\frac{C.T}{log\frac{N}{N_{0}}\times 3.31}$$

### ASCs characterization by flow cytometry

For immunostaining, the cells were defreezed and washed with PBS by centrifuging at 1,200 rpm for 5 min. Then, about 1×10^6^ cells at passage 2 were suspended in a 20µl medium containing 3% FBS (FBS; Sigma, USA). Appropriate fluorochorome conjugated Anti-human antibodies, including human CD31/PE-1PerCP (FAB3567c, R&D), CD45-PerCP (557513, BD), CD90-PE (555596, BD), CD44-FITC, (560977, BD) was added to each tube. The tubes were incubated at 4°C for 20 min in a dark environment, and, then, centrifuged at 1,200 rpm for 5 min. The supernatant was discarded, and 300µl PBS was added to all the tubes. Cell surface antigens were analyzed by flow cytometry at passage 1 and 6.

### Cell adhesion evaluation

The Polycaprolactone (PCL) was sterilized under Ultraviolet (UV) light for 30 min on both sides. About 1×10^5^ cells were seeded on PCL at passage 2. The ASCs-PCL was incubated at 37 °C and 5.0% CO_2_ for 12hr. Samples were coated with Gold. Then, ASCs- PCL construct were evaluated by Scanning Electron Microscopy (Mira3 Te Scan, Czech Republic).

### Statistical analysis

Data was obtained using the flowing software version -2-5-1 and Dot plot from stained samples. SPSS Inc for statistical significance by independent T-Test and Leven′s analysis was performed. Also statistical analysis Cell doubling time was analyzed by one-way analysis of ANOVA test. Data are expressed as p<0.05 were considered statistically significant.

## Results

### Culture of harvested cells

**Figure 1:**
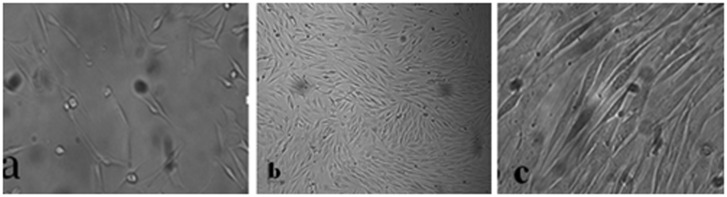
Morphology of ASCs at primary culture (a), confluency after 5 days (b) and morphology of cells at passage 6 (c), studied with invert microscopic.

In the culture of harvested cells from infrapatellar fat pad of sheep, cells displayed fibroblast-like morphology at different subcultures and approached confluency after 5 days (Figure 1).

### Population doubling time in isolated cells

**Figure 2:**
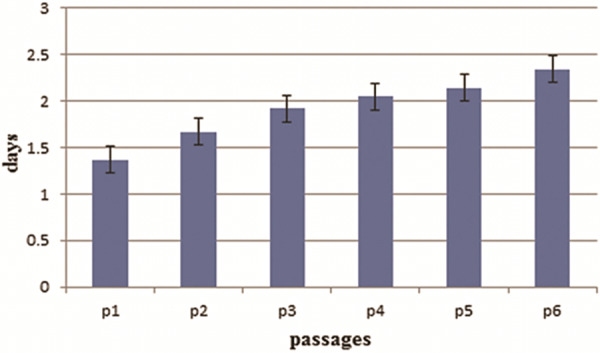
Doubling time of ASCs during continuous passages 1-6.

Cell proliferation rate was determined in isolated ASCs. Population doubling time (PDT) was calculated for every passage (Figure 2). According to data, there was no significant difference between the cell proliferations in passages through 1 to 6.

### Evaluation of ASCs by flow cytometry

**Figure 3:**
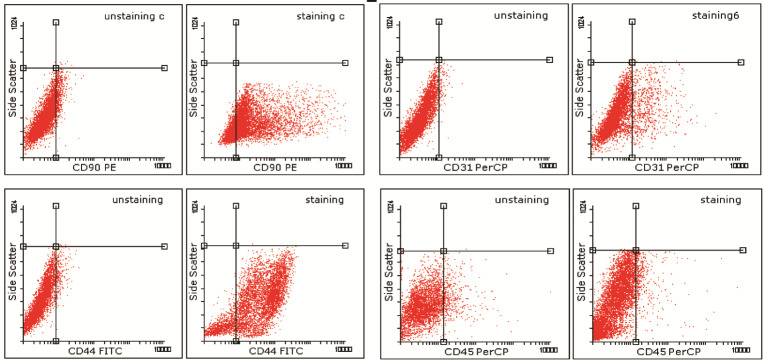
Dot plots of subclass control (unstaining) and CD44 FITC (positive91.84%)/CD90 PE
(Positive 90.5%) and negative for CD31 and CD45 markers at passage 1.

The cell surface markers were evaluated by flow cytometric analysis using anti-human antibody. Harvested cells expressed positive for CD44 (91.84%) and for CD90 (90.5%), and negative for CD31 and Cd45 markers (Figure 3).

**Figure 4:**
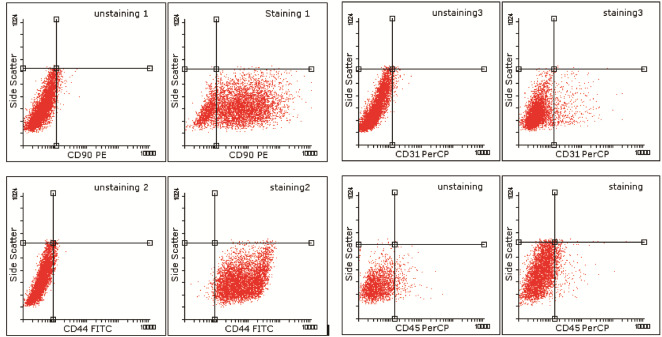
Dot plots of subclass control (unstaining) and CD44 FITC (positive 97.32%)/CD90 PE (Positive 96.86%) and negative for CD31 and CD45 markers at passage 6.

Flow cytometry analysis indicated that the ASCs maintain their cell surface markers at different periods of cell culture. They were positive (97.32%) for CD44 and Positive (96.86%) for CD90 by Anti-Human antibody and negative for CD31 and Cd45 markers (Figure 4) at passage 6.

Statistical analysis did not reveal a significant difference between the behaviors of ASCs surface markers at passages 1 to 6.

### Isolation of ASCs integrated with PCL

**Figure 5:**
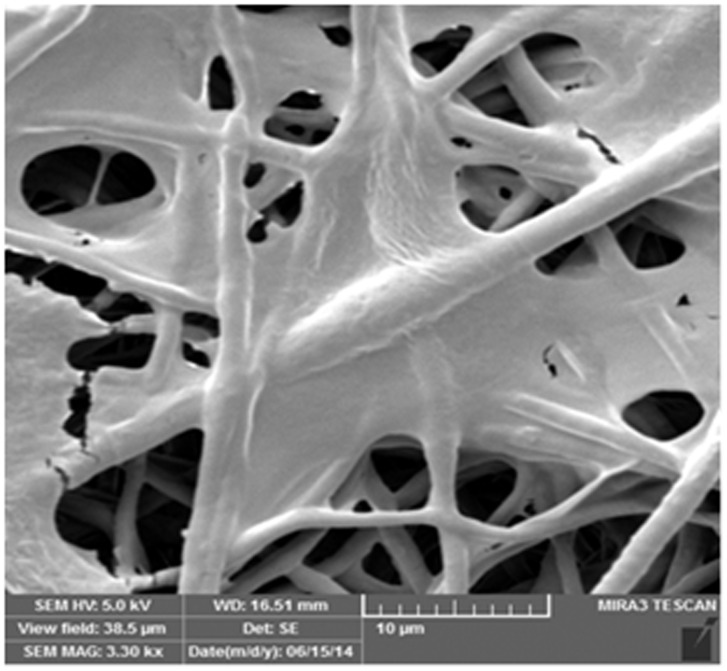
Scanning electron micrograph of cell adhesion on nanofiber scaffold 1/38 Kx.

**Figure 6:**
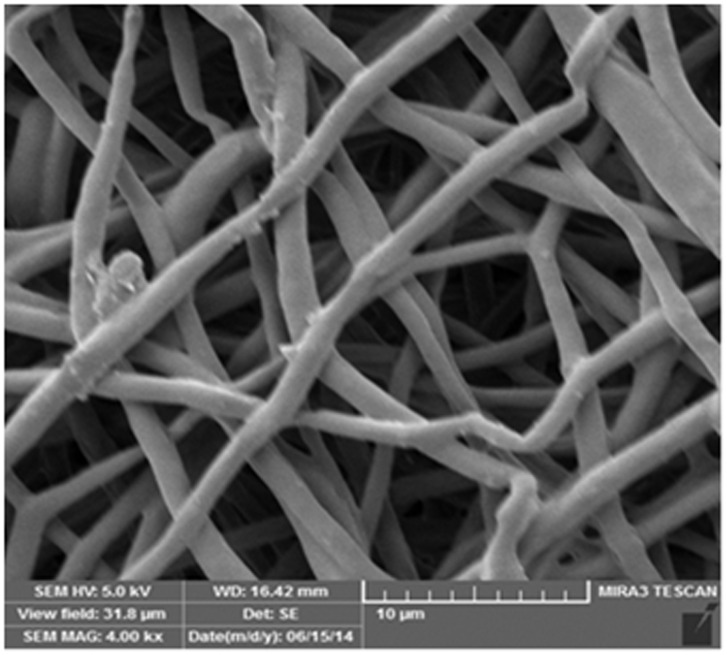
SEM photo micrograph of the nanofiber scaffolds before cell seeding.

Photomicrographs of SEM showed that the adipose tissue derived stem cells of infrapatellar fat pad in sheep have special adherence on nanofiber scaffolds (Figure 5). Nanofiber scaffolds before cell culture have been displayed in (Figure 6).

## Discussion

Results of this study indicated that infrapatellar fat pad adipose tissue derived stem cells maintain fibroblast-like morphology, cell specific adhesion, and test strongly positive for CD90 and CD44. Researchers have isolated MSCs from various tissues such as adipose tissue, umbilical cord blood, peripheral blood, liver, skeletal muscle and dermis.^11,12^ They have mainly focused on isolation of mesenchymal stem cells from adipose tissue in large animal model, due to the fact that they are accessible with ease and have abundant cells available.^13^ Buckley et al. have stated that obtained ASCs from infrapatellar fat pad adipose tissue possess high chondrogenesis potential. However, they have reported that proliferation and differentiation potential of MSCs decreases during several passages. There are different reports on mesenchymal stem cells senescence^14,15^ and, besides, doubling time of ASCs differs between 2 to 5 days; it depends on passage number and culture condition.^16^

Some previous studies have shown that the doubling time of ASCs differs according to the location of adipose tissue.^17^ Others have reported that MSCs obtained from infrapatellar fat pad keep their differentiation potential in the later stages of life.^18^ It was stated that proliferation capacity of ASCs is greater than that of bone marrow-derived stem cells.^19^ Many studies have attempted to characterize ASCs surface markers by flow cytometry analysis. They suggested that CD90 of ASCs expresses less and hasn’t been completely explored in sheep model. But it was expressed in humans^20^ and, yet, characterization of the phenotype of MSCs from different animal species remains a problem due to the lack of species-specific antibodies. There are more selective species-specific antibodies for using in small animals, contrasted with less species-specific antibodies for large animals.^21^ This is mostly because, there are no species-specific antibodies to define MSCs surface markers in large animal models. The studies have also shown that sheep is appropriate for orthopedic research since it has similarities with humans in size, weight, bone/cartilage regenerative processes and joint structure.^22^ Also ample evidence suggests that ovine knee may be accepted as a model for substitution of the human knee in diagnostic practical studies.^23^

Although, human MSC properties have been thoroughly investigated, very little characterization of sheep mesenchymal stem cells has been undertaken. As mentioned earlier, the positive expression of CD90, CD105 and CD73 has been strongly shown in human mesenchymal stem cells. However, they are not displayed by all species. The expression of CD90 has been tested in the majority of species, but it has been absent for mesenchymal stem cells in sheep and goats.^24^ Our findings indicated that infrapatellar fat pad adipose tissue derived stem cells expressed cell surface markers and tested strongly positive for CD90 markers and CD44. Besides, they maintain their fibroblastic-like morphology. Therefore, it is necessary to establish a standard protocol for isolation and characterization of adipose tissue stem cells (ACSs), since it bears many advantages for cell therapy and tissue engineering.^25^

## Conclusion

The results of this study clearly showed that ASCs obtained from infrapatellar fat pad adipose tissue maintain proliferation potential, their fibroblastic- like morphology and their cell surface markers during different passages. So adipose tissue derived stem cells from infrapatellar fat pad are one of the best cell source for tissue engineering.

## Acknowledgments

The authors are sincerely grateful to research department of Tabriz University of Medical Sciences for their financial support. Grant code: 92/4-3/5

## Ethical Issues

Not applicable.

## Conflict of Interest

The authors report no conflicts of interest.
